# Beyond the Classic Triad: A Case of GQ1b Antibody-Positive Bickerstaff Brainstem Encephalitis Overlap Syndrome

**DOI:** 10.7759/cureus.116491

**Published:** 2026-09-18

**Authors:** Jarod T Ormsby, Ajay McLaughlin, Antoni Wojtkowski, Gabriel Zaitta, Taryn Houghton

**Affiliations:** 1 Internal Medicine, East Tennessee State University, Johnson City, USA; 2 Critical Care Medicine, Bristol Regional Medical Center, Bristol, USA; 3 Internal Medicine, Edward Via College of Osteopathic Medicine, Blacksburg, USA

**Keywords:** anti-gq1b antibody, bickerstaff brainstem encephalitis, gbs variant, guillain-barré syndrome, miller fisher syndrome

## Abstract

Anti-GQ1b antibody syndrome encompasses related postinfectious neurologic disorders, including Miller Fisher syndrome, Guillain-Barré syndrome, and Bickerstaff brainstem encephalitis. Recognizing overlap among these phenotypes can be difficult in the intensive care unit because profound unresponsiveness and loss of brainstem reflexes may be attributed to sedation or structural brain injury. A middle-aged man developed perioral numbness, blurred vision, ataxia, extremity paresthesias, and facial weakness after an upper respiratory infection. His condition progressed rapidly to respiratory failure, autonomic instability, generalized areflexia, and prolonged coma-like unresponsiveness. Brain imaging remained unrevealing. Cerebrospinal fluid showed albuminocytologic dissociation, and serum testing was positive for anti-GQ1b antibodies. He received intravenous immunoglobulin and intensive supportive care. Ocular and distal motor responses later returned, followed by resolution of the coma-like state, although ventilatory support was still required at transfer. This case demonstrates that severe anti-GQ1b overlap can produce profound but potentially reversible neurologic dysfunction and supports cautious neuroprognostication while the diagnostic evaluation is ongoing.

## Introduction

Anti-GQ1b antibody syndrome describes a postinfectious autoimmune spectrum that includes Miller Fisher syndrome (MFS), Bickerstaff brainstem encephalitis (BBE), and phenotypes that overlap with Guillain-Barré syndrome (GBS). Anti-GQ1b antibodies bind gangliosides that are concentrated in ocular motor nerves, muscle spindle afferents, peripheral nerves, and possibly the brainstem reticular formation. This shared immunologic target helps explain why central and peripheral findings may occur in the same patient [1-3].

MFS is classically defined by ophthalmoplegia, ataxia, and areflexia, whereas impaired consciousness or pyramidal findings support BBE. Limb weakness and respiratory failure indicate additional GBS involvement [2-5]. These boundaries are clinically useful but incomplete; patients may evolve from one phenotype to another over several days. As brain magnetic resonance imaging (MRI) may remain normal despite severe brainstem dysfunction, diagnosis depends on the clinical pattern and exclusion of structural, toxic, metabolic, and infectious causes, with support from cerebrospinal fluid and ganglioside antibody testing [2,4,6].

True MFS-BBE-GBS overlap is uncommon, and published reports show substantial variation in neuroimaging, duration of mechanical ventilation, response to immunotherapy, and pace of recovery [5,7]. Severe overlap accompanied by prolonged coma-like unresponsiveness and near-total loss of brainstem reflexes is particularly difficult to distinguish from residual sedation or irreversible neurologic injury. Documenting this phenotype clarifies the diagnostic value of serial examinations and supports cautious prognostication during continued intensive care.

## Case presentation

A 43-year-old male with a history of poorly controlled hypertension presented to the emergency department after five hours of sudden-onset perioral numbness, blurred vision, ataxia, bilateral extremity paresthesias, and facial weakness. Four days earlier, he had developed an upper respiratory infection for which he was taking amoxicillin and benzonatate. His initial blood pressure was 229/115 mmHg. Examination showed bilateral facial nerve weakness and gait ataxia. The abrupt onset, cranial and bulbar findings, and severe hypertension prompted evaluation for posterior circulation ischemia, hypertensive encephalopathy or posterior reversible encephalopathy syndrome, myasthenia gravis, and botulism. The preceding infection, ataxia, paresthesias, and evolving cranial neuropathies also raised concern for an MFS, BBE, or GBS-spectrum disorder.

Initial computed tomography and brain MRI showed no acute ischemia or other intracranial process. Within 24 hours of admission, he developed tachypnea, tachycardia, severe hypoxemia with oxygen saturation in the 70s, and unresponsiveness. He required endotracheal intubation and prolonged mechanical ventilation for acute hypoxic respiratory failure. Thick endobronchial secretions required aggressive pulmonary hygiene and multiple therapeutic bronchoscopies. His course was further complicated by marked hemodynamic lability requiring vasopressor support, acute rhabdomyolysis with a peak creatine kinase of 4,566 U/L on hospital day six, and acute kidney injury with a peak serum creatinine of 2.64 mg/dL.

Neurologic examination showed generalized areflexia and near-complete loss of brainstem reflexes. Corneal, gag, cough, and vestibulo-ocular reflexes were absent; sluggish pupillary reactivity was the only preserved brainstem reflex. Cerebrospinal fluid was colorless with a protein concentration of 62 mg/dL and 3 white blood cells/µL, consistent with albuminocytologic dissociation. Electroencephalography showed moderately severe encephalopathy. Repeat brain MRI without contrast showed no acute intracranial process. A serum ganglioside antibody panel was positive for anti-GQ1b IgG/IgM antibodies at a titer of 360. The combination of oculomotor and bulbar involvement, ataxia, generalized areflexia, respiratory failure, and impaired consciousness supported an anti-GQ1b-positive MFS-BBE-GBS overlap syndrome.

The patient received a five-day course of intravenous immunoglobulin (IVIg) together with mechanical ventilation, vasopressor support, pulmonary hygiene, and other organ-directed care. After continuous propofol and fentanyl infusions were discontinued, he remained profoundly unresponsive for eight days. Serial examinations later identified minimal voluntary bilateral toe movement and return of ocular movement on hospital days 19-20. At that time, he remained unable to move his head, tongue, or limbs otherwise to command.

By hospital day 30, the coma-like unresponsiveness had resolved, and focal motor function was improving. Renal function returned to baseline, with serum creatinine decreasing to 0.51 mg/dL, and blood pressure stabilized on oral antihypertensive therapy. He underwent tracheostomy and was transferred to a long-term acute care hospital for continued ventilator management and rehabilitation. At the last documented inpatient follow-up before transfer, he was alert and cooperative and was regaining peripheral strength but remained ventilator-dependent through the tracheostomy.

## Discussion

This patient’s combination of ataxia and cranial neuropathies, generalized areflexia and respiratory failure, and prolonged impaired consciousness is most consistent with an overlap of MFS, GBS, and BBE rather than an isolated phenotype [2,3,5]. Anti-GQ1b seropositivity and albuminocytologic dissociation supported this classification. The normal serial MRIs did not exclude BBE; structural abnormalities are absent in many reported cases, and the diagnosis remains primarily clinical [2,4].

The published cases summarized in Table 1 show why the present course is diagnostically important. Puma et al. described an anti-GQ1b-positive triple-overlap syndrome with normal serial MRI, coma, tetraplegia, and ventilator dependence, but ventilator liberation occurred later than the first objective recovery in our patient [8]. Natsui et al. likewise reported anti-GQ1b-positive BBE after an upper respiratory illness with normal serial MRI, deep coma, and mechanical ventilation; the patient followed commands earlier but required ventilation until hospital day 29 [9]. Wang et al. reported BBE-GBS overlap with coma and tetraparesis, but MRI showed a medullary lesion and near-complete recovery required 10 months [10].

**Table 1:** Comparison with selected published BBE and anti-GQ1b overlap cases. BBE = Bickerstaff brainstem encephalitis; GBS = Guillain-Barré syndrome; IVIg = intravenous immunoglobulin; MFS = Miller Fisher syndrome; MRI = magnetic resonance imaging; URI = upper respiratory infection

Report	Diagnostic parallels	Key difference	Reported outcome
Present case	Post-URI; MFS-BBE-GBS features; anti-GQ1b positive; albuminocytologic dissociation; normal serial MRI; coma-like unresponsiveness; mechanical ventilation	Near-total loss of brainstem reflexes persisted after sedation stopped; pupils remained sluggishly reactive	Toe and ocular movement returned on days 19-20; coma-like unresponsiveness resolved by day 30; ventilator-dependent at transfer
Puma et al. [8]	Triple overlap; anti-GQ1b positive; normal serial MRI; coma, tetraplegia, and mechanical ventilation	*Mycoplasma pneumoniae* trigger; persistent conduction blocks; multiple IVIg courses	Ventilator weaning on day 41; rehabilitation transfer on day 57; continued motor recovery through day 240
Natsui et al. [9]	Post-URI BBE; anti-GQ1b positive; normal serial MRI; deep coma and mechanical ventilation	*Haemophilus influenzae* isolated; initially treated for possible viral or autoimmune encephalitis	Followed commands on day 13; ventilator weaning on day 29; rehabilitation transfer on day 65
Wang et al. [10]	BBE-GBS overlap with cranial neuropathies, ataxia, tetraparesis, coma, and albuminocytologic dissociation	MRI showed a T2-hyperintense medullary lesion rather than normal imaging	Near-complete recovery at 10 months

Across these reports, the recurring combination of a preceding infection, oculomotor or bulbar dysfunction, impaired consciousness, peripheral areflexia or weakness, and supportive anti-GQ1b or cerebrospinal fluid findings indicates a continuous central-peripheral disease spectrum. The present case most closely resembles the normal-imaging, ventilated courses of Puma et al. and Natsui et al. [8,9]. Its distinguishing feature was the persistence of an extremely poor brainstem examination after sedatives were stopped, followed by delayed but objective neurologic recovery. In contrast, the medullary MRI abnormality in Wang et al. shows that imaging findings vary and that neither a normal nor abnormal MRI determines reversibility [10].

The change from severe hypertension at presentation to vasopressor-dependent hemodynamic instability is compatible with dysautonomia in a GBS-spectrum illness, although critical illness, sedatives, and respiratory failure were potential contributors. This distinction matters because autonomic lability, respiratory weakness, impaired airway protection, and secretion burden can prolong intensive care even after consciousness begins to improve. In this patient, neurologic recovery preceded ventilator independence, which parallels the dissociation between awakening and later ventilator liberation reported by Natsui et al. [9].

Evidence for BBE-specific immunotherapy remains limited to observational series and case reports. IVIg and plasma exchange are commonly selected by extrapolation from GBS treatment guidelines [2,11]. Our patient’s improvement followed IVIg, but a single case cannot establish that treatment caused or accelerated recovery. The defensible clinical lesson is therefore diagnostic and supportive: when profound unresponsiveness, cranial neuropathies, areflexia, and respiratory failure occur after an infection, normal neuroimaging should not end the evaluation. Serial neurologic examinations, cerebrospinal fluid analysis, ganglioside antibody testing, and continued organ support can reveal a potentially reversible anti-GQ1b overlap syndrome.

## Conclusions

In this patient, anti-GQ1b-positive MFS-BBE-GBS overlap produced prolonged coma-like unresponsiveness, near-total loss of brainstem reflexes, dysautonomia, and respiratory failure despite normal neuroimaging. Delayed return of ocular and distal motor responses preceded resolution of the coma-like state. Clinicians should maintain supportive care and avoid premature neurologic prognostication while serial examinations and targeted testing clarify the diagnosis.

## References

[1] Odaka M, Yuki N, Hirata K (2001). Anti-GQ1b IgG antibody syndrome: clinical and immunological range. J Neurol Neurosurg Psychiatry.

[2] Shahrizaila N, Yuki N (2013). Bickerstaff brainstem encephalitis and Fisher syndrome: anti-GQ1b antibody syndrome. J Neurol Neurosurg Psychiatry.

[3] Wakerley BR, Uncini A, Yuki N (2014). Guillain-Barré and Miller Fisher syndromes--new diagnostic classification. Nat Rev Neurol.

[4] Odaka M, Yuki N, Yamada M, Koga M, Takemi T, Hirata K, Kuwabara S (2003). Bickerstaff's brainstem encephalitis: clinical features of 62 cases and a subgroup associated with Guillain-Barré syndrome. Brain.

[5] Sekiguchi Y, Mori M, Misawa S, Sawai S, Yuki N, Beppu M, Kuwabara S (2016). How often and when Fisher syndrome is overlapped by Guillain-Barré syndrome or Bickerstaff brainstem encephalitis?. Eur J Neurol.

[6] Nishimoto Y, Odaka M, Hirata K, Yuki N (2004). Usefulness of anti-GQ1b IgG antibody testing in Fisher syndrome compared with cerebrospinal fluid examination. J Neuroimmunol.

[7] Coly M, Adams D, Attarian S (2024). Clinical, paraclinical and outcome features of 166 patients with acute anti-GQ1b antibody syndrome. J Neurol.

[8] Puma A, Benoit J, Sacconi S, Uncini A (2018). Miller Fisher syndrome, Bickerstaff brainstem encephalitis and Guillain-Barré syndrome overlap with persistent non-demyelinating conduction blocks: a case report. BMC Neurol.

[9] Natsui H, Takahashi M, Nanatsue K, Itaya S, Abe K, Inaba A, Orimo S (2021). Mother and son cases of Bickerstaff's brainstem encephalitis and Fisher syndrome with serum anti-GQ1b IgG antibodies: a case report. BMC Neurol.

[10] Wang DS, Tang Y, Wang Y (2006). A case of overlapping Bickerstaff's brainstem encephalitis and Guillain-Barré syndrome. J Zhejiang Univ Sci B.

[11] van Doorn PA, Van den Bergh PY, Hadden RD (2023). European Academy of Neurology/Peripheral Nerve Society Guideline on diagnosis and treatment of Guillain-Barré syndrome. Eur J Neurol.

